# Supplementary material for: Insights on the Biomarker Potential of Exosomal Non-Coding RNAs in Colorectal Cancer: An In Silico Characterization of Related Exosomal lncRNA/circRNA–miRNA–Target Axis
Source: Cells. 2023 Apr 4;12(7):1081. doi: 10.3390/cells12071081 (PMC10093117; doi:10.3390/cells12071081)
Supplement: Supplementary file 1 [file cells-12-01081-s001.zip › cells-2211591-supplementary.pdf]

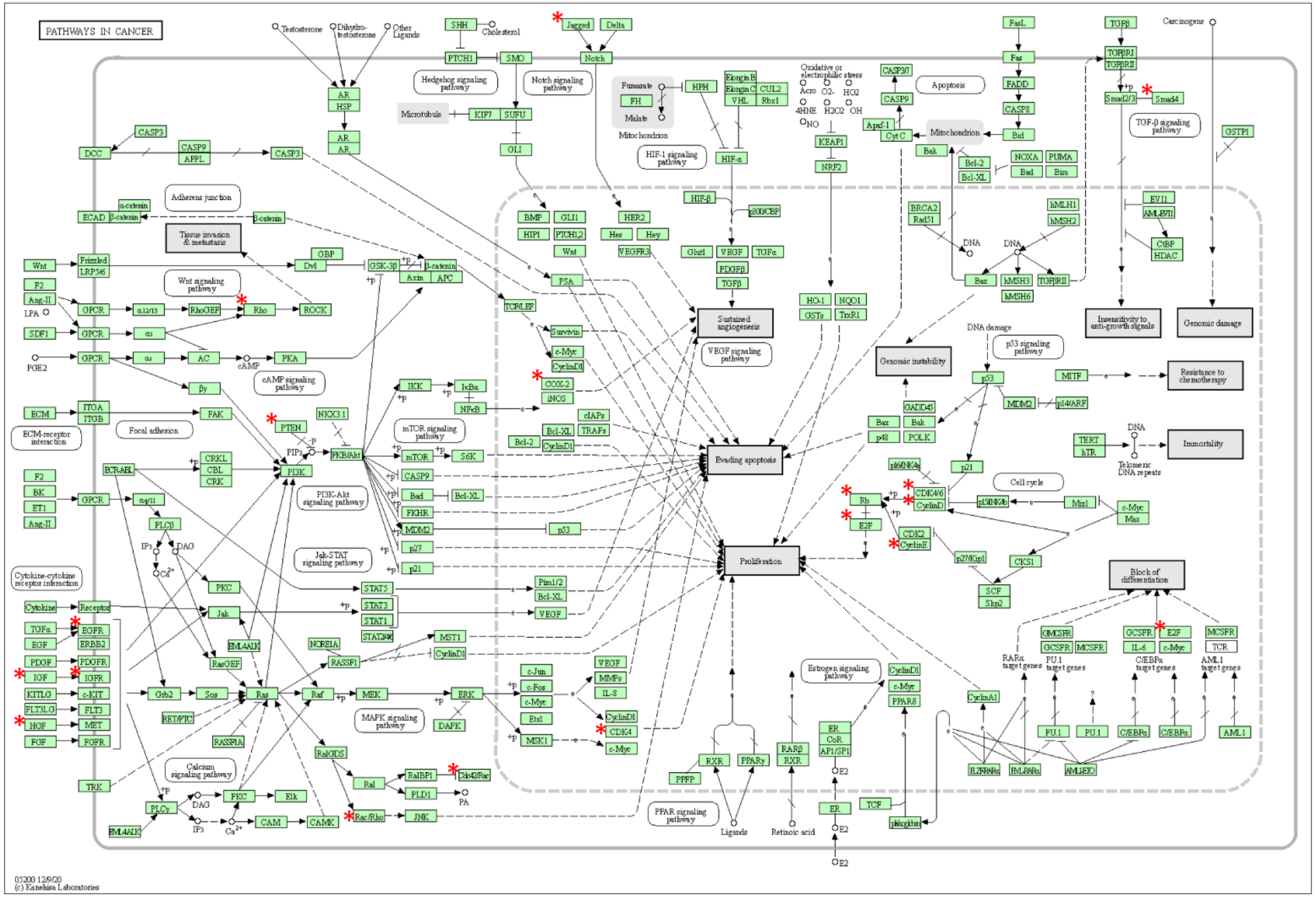

Figure S1. Implication of the strong experimentally validated shared target genes downstream of the CRC exosomal lncRNA/circRNA-miRNA-target axis in Pathways in Cancer KEGG pathway. 16 out of the 38 shared target genes were found to be implicated in Pathways in Cancer KEGG pathway. Red stars indicate the shared target genes.

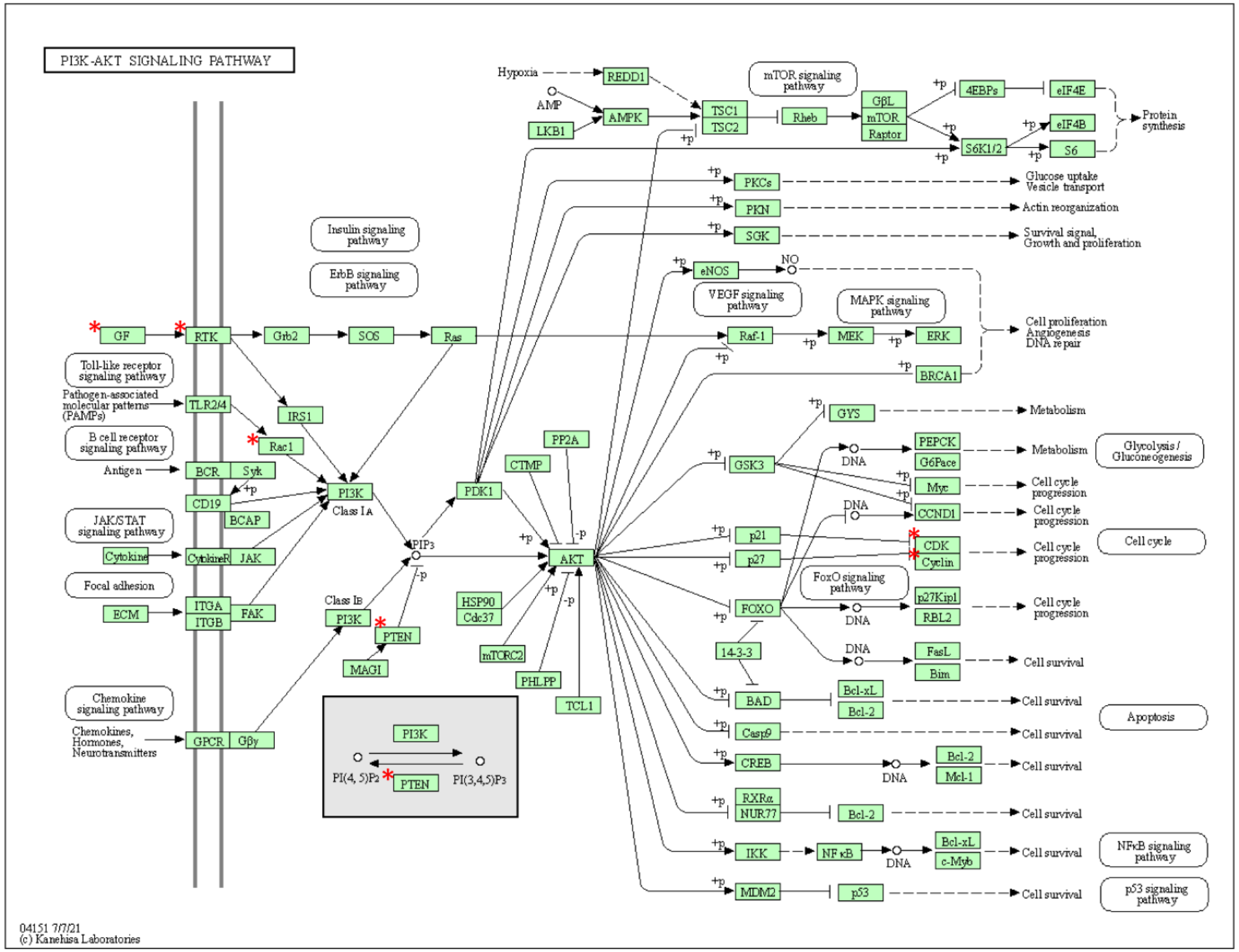

Figure S2. Implication of the strong experimentally validated shared target genes downstream of the CRC exosomal lncRNA/circRNA-miRNA-target axis in PI3K-Akt signaling KEGG pathway. 10 out of the 38 shared target genes were found to be implicated in PI3K-Akt singlaing KEGG pathway. Red stars indicate the shared target genes.
